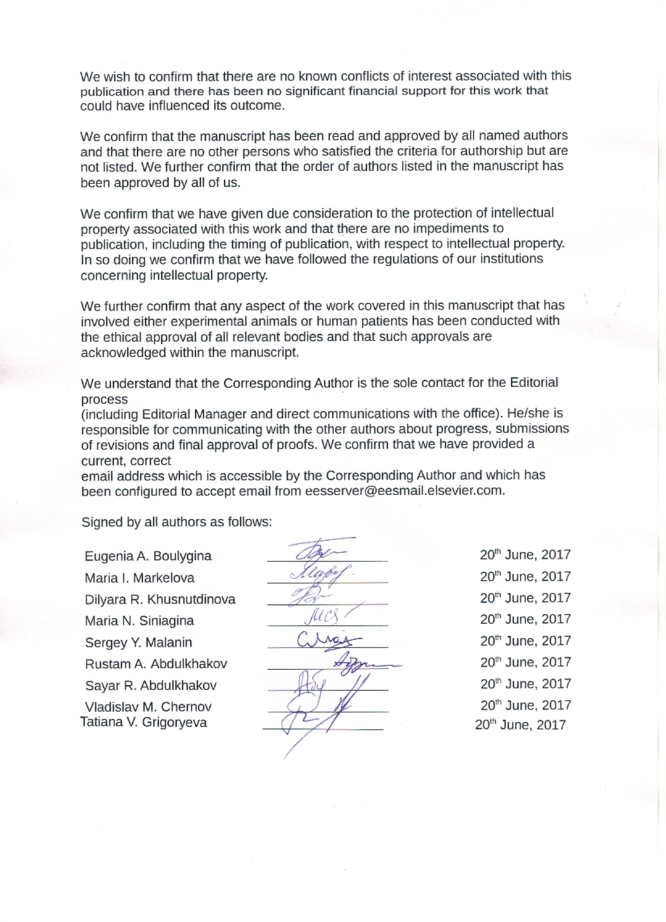# Shotgun metagenomic data on the human stool samples to characterize shifts of the gut microbial profile after the *Helicobacter pylori* eradication therapy

**DOI:** 10.1016/j.dib.2017.07.070

**Published:** 2017-07-28

**Authors:** Eugenia A. Boulygina, Maria I. Markelova, Dilyara R. Khusnutdinova, Maria N. Siniagina, Sergey Yu. Malanin, Rustam A. Abdulkhakov, Sayar R. Abdulkhakov, Vladislav M. Chernov, Tatiana V. Grigoryeva

**Affiliations:** a Kazan Federal University, 18 Kremlyovskaya, 420008 Russia; b Kazan State Medical University, 49 Butlerova, 420012 Russia

**Keywords:** Human gut microbiota, *Helicobacter pylori*, Eradication, Antibiotics

## Abstract

The shotgun sequencing data presented in this report are related to the research article named “Gut microbiome shotgun sequencing in assessment of microbial community changes associated with *H. pylori* eradication therapy” (Khusnutdinova et al., 2016) [1]. Typically, the *H. pylori* eradication protocol includes a prolonged two-week use of the broad-spectrum antibiotics. The presented data on the whole-genome sequencing of the total DNA from stool samples of patients before the start of the eradication, immediately after eradication and several weeks after the end of treatment could help to profile the gut microbiota both taxonomically and functionally. The presented data together with those described in Glushchenko et al. (2017) [2] allow researchers to characterize the metagenomic profiles in which the use of antibiotics could result in dramatic changes in the intestinal microbiota composition. We perform 15 gut metagenomes from 5 patients with *H. pylori* infection, obtained through the shotgun sequencing on the SOLiD 5500 W platform. Raw reads are deposited in the ENA under project ID PRJEB21338.

**Specifications Table**

**Table:** 

Subject area	*Biology*
More specific subject area	*Metagenomics*
Type of data	*DNA sequences*
How data was acquired	*Shotgun DNA sequencing using SOLiD 5500 W platform*
Data format	*Raw*
Experimental factors	*Total DNA extraction from stool samples*
Experimental features	*Single-end read libraries were created from 5 μg of total DNA. Shotgun metagenomic sequencing was performed on SOLiD 5500 W platform according to the recommendations of the manufacturer.*
Data source location	*Kazan, Russian Federation*
Data accessibility	*Data are available from ENA under project accession number PRJEB21338*

**Value of the data**

• The data allow researchers to evaluate changes in the taxonomic and functional composition of the human gut microbiota, which is associated with the use of amoxicillin and clarithromycin.
• Since the data include the metagenome profile at the 3rd time point (several weeks after the end of *H. pylori* eradication), it is possible to make assumptions about the reversibility/irreversibility of therapy-related changes.
• The data can be used to estimate the distribution of antibiotic resistance genes in the genetic pool of the gut microbiota after *H. pylori* eradication therapy.
• Using this data one can describe the general trends of the microbial composition variation caused by antibiotics and predict possible side effects.
• The data can be used to detect the genetic markers of dysbiosis and to design a minimally invasive PCR diagnostic system.

## 1 Data

The data represent the result of metagenomic shotgun-sequencing of human gut microbiota at 3 time points: before the *H. pylori* eradication therapy, immediately after 2 weeks of therapy and several weeks after the treatment. The dataset contains 15 metagenomic samples in raw reads format with 30.4 ± 10.7 mln of reads per sample (mean ± SD).

These data together with those described in [2] were involved in the study devoted to the gut microbiome changes caused by antimicrobial therapy [1].

These data together with those described in [2] were involved in the study devoted to the gut microbiome changes caused by antimicrobial therapy [1].

Detailed description of samples is given in Table 1.

**Table 1:** Description of samples. “Time point” column shows the time of sample collection: “1” means at the start of the therapy, “2” – immediately after therapy, “3” – several weeks after therapy.

Patient ID	Gender	Diagnosis	Sample ID	Time point
1	Male	Gastroesophageal reflux disease	1HP	1
			2HP	2
			3HP	3 (3.5 weeks after treatment)
				
2	Male	Chronic gastroduodenitis	26HP	1
			27HP	2
			28HP	3 (4 weeks after treatment)
				
3	Female	Gastroesophageal reflux disease	41HP	1
			42HP	2
			43HP	3 (11 weeks after treatment)
				
4	Female	Chronic gastroduodenitis	31HP	1
			53HP	2
			54HP	3 (4 weeks after treatment)
				
5	Male	Gastroesophageal reflux disease	63HP	1
			64HP	2
			65HP	3 (2.5 weeks after treatment)

## 2 Experimental design, materials and methods

### 2.1 Sample collection

Fifteen stool samples from 5 patients with symptoms of stomach and/or duodenum disease and *Helicobacter pylori* detected by endoscopy were taken for analysis. According to the Maastricht V Consensus and recommendations of Russian Gastroenterological Association, patients had been prescribed a two-week eradication therapy consisted of amoxicillin (1000 mg bid), clarithromycin (500 mg bid) and proton pump inhibitors at a standard dose accompanied by bismuth subsalicylate (240 mg bid) [3]. Samples were collected in the Polyclinic Department of the Republican Clinical Hospital #2 (Kazan). The study was approved by the Local Ethics Committee of the Kazan Federal University. Prior written informed consent was obtained from all patients.

### 2.2 Sample preparation and sequencing

Fecal samples were collected in the individual plastic containers, avoiding contamination with urine or toilet tissue. 10–20 g of samples were instantly frozen with subsequent storage at −20 °C, or were used for DNA extraction immediately after sampling.

Total DNA (5 μg) from fecal samples was extracted using the Purelink Genomic DNA Extraction Kit (Invitrogen, USA). Metagenomic single-end library preparation and shotgun sequencing on SOLiD 5500 W platform was performed according to the recommendations of the manufacturer (LifeTechnologies, USA). The resulting color-spaced reads with length of 50 bp were converted to the base-space format.

## Figures and Tables

**Fig. S1:**